# RNA modifications in gynecological cancer: current status and future directions

**DOI:** 10.3389/fmed.2024.1314075

**Published:** 2024-01-26

**Authors:** Wanshan He, Xiaoshan Hong, Guanqiao Chen, Xiping Luo, Yu Lin

**Affiliations:** Department of Gynecology, Guangdong Women and Children Medical Hospital, Guangzhou, China

**Keywords:** gynecological cancer, RNA modification, epigenetics, N6-methyladenosine, 5-methylcytosine

## Abstract

Currently, more than 170 modifications have been identified on RNA. RNA modification mainly regulates RNA splicing, intracellular transport, degradation, translation, and stability. Gynecologic cancer (GC) mainly includes cervical cancer (CCA), ovarian cancer (OC), Endometrial cancer (EMC), among others, is the leading cause of cancer-related death. At present, there is still a lack of effective means to eradicate such diseases, so it is important to conduct more in-depth research on gynecological cancers. Numerous studies have shown that a series of epigenetic changes occur during the development of gynecologic cancer. This article reviews the latest findings on the functional significance of RNA modification in gynecologic cancer and discusses the therapeutic potential of RNA modification-related inhibitors in the treatment of gynecologic cancer.

## 1 Introduction

Epigenetics is a discipline that specializes in the study of heritable gene expression or cell phenotypic changes without changing the nucleotide sequence of inherited genes (1), and known epigenetic phenomena include DNA methylation, RNA modification, protein acetylation, protein methylation (1–3). Among them, RNA modification has been a research hotspot in the field of epigenetics in recent years, and more than 170 types of RNA modification have been identified in organisms (4). Among these RNA modification types, the main ones are N6-methyladenosine(m^6^A),5-methylcytosine(m^5^C), N1-methyladenosine(m^1^A), N7-Methylguanosine(m^7^G), Pseudouridine(ψ) and adenosine-to-inosine(A-to-I) editing modifications (5). More and more research data show that RNA modification plays an important role in the occurrence and progression of cancer, such as breast cancer (6), liver cancer (7), bladder cancer (8), lung cancer (9). Gynecological cancers mainly include vulvar cancer, vaginal cancer, cervical cancer, uterine body cancer, ovarian cancer, of which cervical cancer, uterine body cancer and ovarian cancer are the three most common types of gynecological cancer.

According to the 2020 Global Cancer Statistics Report, around 1.4 million women were diagnosed with gynecological cancers in 2020, resulting in approximately 671,875 deaths (10). This imposes a significant burden on women worldwide, both physically and psychologically. Although current treatment methods for gynecological cancer primarily involve surgery, radiotherapy, and chemotherapy, these approaches still fail to completely address issues such as metastasis and recurrence. The current development of medical technology still has no effective way to cure gynecological cancer, therefore, it is necessary and urgent to further deepen the research on the unknown mechanism of gynecological cancer. Here, we summarize the recent research progress of RNA modification in the field of gynecological cancer, to provide new ideas for the early diagnosis and treatment of gynecological cancer.

## 2 m^6^A modification in gynecological cancer

m^6^A is the most common internal modification mode of eukaryotic mRNA, which is the methylation of adenosine at the 6th position (11). m^6^A methyltransferase (writer) mediates methylation modification on mRNA through m^6^A, while demethylase (eraser) can reverse this modification. Additionally, the m^6^A recognition protein (reader) participates by identifying methylation modification information on mRNA, thereby influencing splicing, degradation, stability, and translation processes (11, 12). Abnormalities in RNA modification processes are closely related to the pathogenesis and progression of gynecological cancers.

### 2.1 Writer

**Figure 1:**
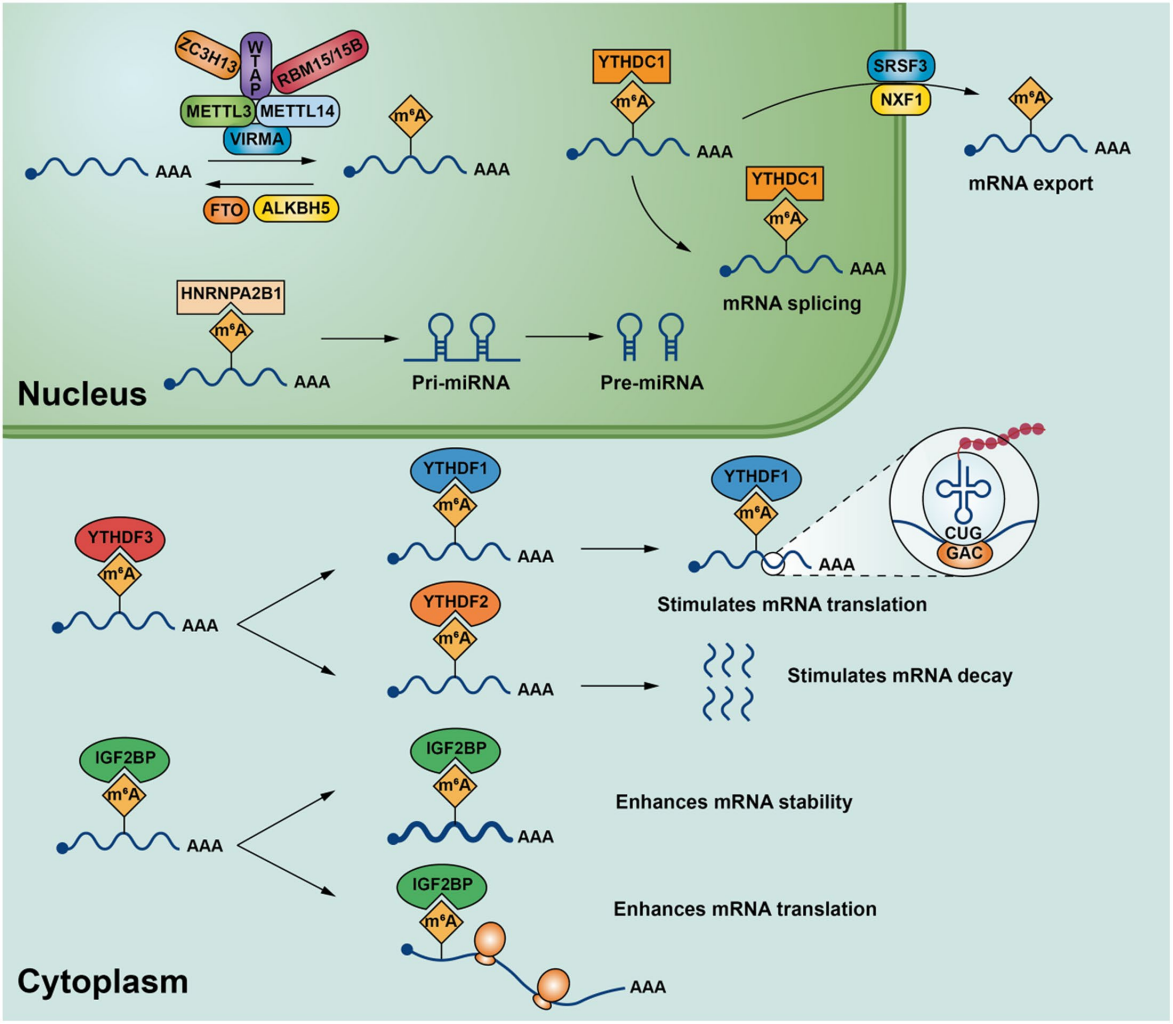
m^6^A modification-related enzymes in mRNA and their major biological functions.

According to current studies, METTL3(methyltransferase-like 3), METTL14(methyltransferase-like 14) (13), WTAP(Wilms’ tumor 1-associating protein) (14), KIAA1429(vir-Like m^6^A methyltransferase associated) (15), RBM15/15B(RNA-binding motif protein 15/RNA-binding motif protein 15B) (16), ZC3H13(Zinc finger CCCH-type containing 13) (17) belong to m^6^A methyltransferase. METTL3 and METTL14 are the core components of m^6^A methyltransferase, and METTL3 is often associated with METTL14 binding forms a stable heterodimeric core complex form involved in the deposition of m^6^A in mammalian nuclear RNA (18). METTL3 it is the only catalytic subunit that binds to the methyl donor S-adenosylmethionine (SAM) and catalyzes methyl transfer, while METTL14 is often required for METTL3 activation and is used to identify substrates RNA plays a key role (13). WTAP is the main component involved in the regulation of the m^6^A methylation complex, which interacts with METTL3 and METTL14,assisting METTL3 and METTL14 are localized to nuclear spots rich in precursor mRNA and is required for the catalytic activity of m^6^A methyltransferase *in vivo* (18). KIAA1429, also known as VIRMA, can recruit METTL3/METTL14/WTAP, the core component of m^6^A to regulate region-selective methylation, VIRMA plays a key role in the specific deposition of m^6^A on 3’UTR (19). Zinc finger protein ZC3H13 also plays an important role in mRNA’s m^6^A methylation modification, which is coordinated with WTAP, Virilizer and Hakai combine to form complexes that allow WTAP to be accurately localized in the nucleus to promote m^6^A methylation (17). RBM15 and/or RBM15B often bind to U-rich sites. During m^6^A modification of mRNA, they bind to WTAP-METTL3 enabling the WTAP-METTL3 complex to bind to specific mRNA and promote further adenosine methylation (16) (Figure 1).

Abnormal modification of m^6^A methyltransferase is associated with the development and progression of gynecologic cancer, and its components have been implicated as both cancer promoters and cancer suppressors. In cervical cancer, METTL3 plays an important role in the different biological processes of cervical cancer by regulating the transcription and translation levels of different oncogenic targets. For example, in the process of cell cycle, METTL3 can enhance the translation of FOXD2-AS1 (lncRNA FOXD2 adjacent opposite strand RNA 1) mRNA, recruit the key transcription silencing factor LSD1 (lysine-specific demethylase 1) to the promoter of p21 to silence the transcription of cell cycle inhibitor p21, thus promoting the proliferation and migration of cervical cancer and inhibiting the apoptosis of cancer cells (20); In addition, CDC25B (cell division cyclin 25B) is an important factor affecting the activation of cyclin-dependent kinases (21). METTL3 can also affect the translation of CDC25B mRNA which will accelerate the process of cell cycle and promote the growth of cervical cancer cells (22). During the aerobic glycolysis of cancer cells, Pyruvate dehydrogenase kinase 4 (PDK4) is one of the most important factors which can direct carbon flux into glycolysis from oxidative phosphorylation (OXPHOS) (23) and HK2 (hexokinase 2) is a key rate-limiting enzyme in aerobic glycolysis (24). The modification of its coding region by METTL3 can enhance its translational efficiency, thereby facilitating glycolysis in cervical cancer and promoting the progression of the disease (25, 26). METTL14, also an oncogene in cervical cancer, enhanced its m^6^A methylation levels by being mediated by piRNA-14633 (PIWI-interacting RNA-14633) to activate the piRNA-14633/METTL14/CYP1B1 axis which promoting the proliferation, invasion and metastasis of cervical cancer cells (27). The importance of the m^6^A pathway in cervical cancer is well established and, as a general effect, m^6^A methylation is necessary to maintain high levels of translation of key transcripts in cervical cancer.

Surprisingly, in ovarian cancer, m^6^A methyltransferase appears to play a contradictory role. It is well known that microRNAs (miRNAs) play an important role in tumor-related diseases (28, 29). Studies have shown that METTL3 can inhibit the expression of PTEN (phosphatase and tensin homolog) by promoting the maturation of pre-miRNA-126-5p (pre-microRNA-126-5p) to deactivate PI3K/Akt/mTOR pathway (30). Or inhibiting CCNG2 (cyclin G2) activity by promoting the maturation of pre-miR-1246 (pre-microRNA-146) (31). Thus, they all ultimately led to the enhancement of the proliferation, migration and invasion ability of ovarian cancer cells, and inhibited cell apoptosis. In addition, VIRMA has also been found to be highly expressed in ovarian cancer tissues, and its expression level is negatively correlated with the survival rate (32, 33). Although the specific mechanism of action has not been clarified, some researchers have considered that VIRMA may be related to the WNT signaling pathway process in ovarian cancer (32). Unlike these two, METTL14 has the opposite effect in ovarian cancer. Overexpression of METTL14 can reduce the mRNA stability of target gene by mediating m^6^A methylation modification which will reduce the expression of trophinin-associated protein (TROAP), thus placing ovarian tumor cells in the G1 phase of the cell cycle and inhibiting their proliferation (34).

In endometrial cancer, METTL3 and METTL14 exhibit a tumor suppressor function. Either the mutation of METTL14 or the downregulation of METTL3 expression can impact on the mRNA stability of PHLPP2 (PH domain and leucine rich repeats protein phosphatase 2) and mTORC2 (Mechanistic target of rapamycin kinase complex 2) that the key members within the AKT pathway which leads to the activation of the AKT signaling pathway and promotes the proliferation, invasion, metastasis, and colony formation of endometrial cancer cells (35).

In summary, m^6^A methyltransferase has important oncogenic and tumor suppressor effects in different types of gynecological cancer. The expression of its major functions is likely to be mainly dependent on the downstream target of its specific action (Table 1).

**Table 1:** Role of RNA epigenetic modifications in gynecologic cancer.

RNA modification	Category	Cancer type	Molecular mechanism	Functional role	Refs
m^6^A					
METTL3	Writer	Cervical cancer	Enhance the translation of FOXD2-AS1 mRNA	Promote the proliferation and migration of cancer cells and inhibit apoptosis	(20)
			Affect the translation of CDC25B mRNA	Accelerate the process of cell cycle and promote the growth of cervical cancer cells	(22)
			Enhance the translation of PDK4 mRNA	Accelerate glycolysis of tumors	(25)
			Enhance the translation of HK2 mRNA	Accelerate glycolysis of tumors	(26)
		Ovarian cancer	Promote the mature targeting of miR-126-5p to inhibit the expression of PTEN, thereby activating the PI3K/Akt/mTOR pathway	Promote the proliferation, migration and invasion of cancer cells, and inhibit apoptosis	(30)
			Promote the mature targeting of miR-1246 to inhibit CCNG2 activity	Promote the proliferation, migration and invasion of cancer cells, and inhibit apoptosis	(31)
		Endometrial cancer	Affect the stability of PHLPP2 and mTORC2 mRNA, which leads to the inhibited of AKT signaling pathway	Inhibit the proliferation, invasion, metastasis and colony formation of cancer cells	(35)
VIRMA	Writer	Ovarian cancer	Unknown	Inversely correlated with survival	(32, 33)
METTL14	Writer	Cervical cancer	activate the piRNA-14633/METTL14/CYP1B1 axis	Promote the proliferation, invasion and metastasis of cervical cancer cells	(27)
		Ovarian cancer	Reduce the stability of TROAP mRNA	Keep ovarian tumor cells in G1 phase of cell cycle and inhibit their proliferation.	(34)
		Endometrial cancer	Affect the stability of PHLPP2 and mTORC2 mRNA	Inhibit the proliferation, invasion, metastasis and colony formation of cancer cells	(35)
FTO	Eraser	Cervical cancer	Enhance the translation of E2F1 and MYC	Promote the proliferation and migration of cancer cells	(42)
		Ovarian cancer	Reduces m6A levels and mRNA transcript stability which inhibits the hydrolysis of the second messenger cAMP mediated	Inhibit the ability of ovarian cancer stem cells to self-renew, proliferate, and spheroid formation	(44)
		Endometrial cancer	Reduce HOXB13 mRNA decay and increases HOXB13 protein expression, thereby activating WNT signaling pathway	Promote tumor metastasis and invasion	(43)
ALKBH5	Eraser	Ovarian cancer	Enhance the stability of bcl-2 mRNA, thereby promoting the interaction between bcl-2 and Beclin1	Inhibit autophagy of EOC and promoting the proliferation and invasion of cancer cells	(46)
		Endometrial cancer	Inhibit the decay of IGF1R transcript and promote the expression of IGF1R, thereby inducing the expression of COL1A1and MMP9	Enhance the proliferation, invasion and migration of endometrial cells	(47)
YTHDF2	Reader	Ovarian cancer	Promote the attenuation of m6A modified transcript, thereby down-regulating the expression level of pro-apoptotic protein BMF	Promote tumor proliferation and inhibit apoptosis	(58)
		Endometrial cancer	promote the degradation of Long noncoding RNA FENDRR, thereby up-regulating the protein expression level of SOX4	Promote the proliferation of cancer cells and inhibit cell apoptosis	(60)
YTHDF1	Reader	Ovarian cancer	promote the translation and overall output of EIF3C mRNA	Promote tumor proliferation, invasion, and metastasis	(61)
IGF2BP1	Reader	Endometrial cancer	Binding to the m6A site of SOX2 mRNA 3’UTR, promote mRNA stability and inhibition of attenuation which enhance the expression of oncogene SOX2	Promote colony formation, invasion and migration of endometrial cancer	(63)
IGF2BP2	Reader	Cervical cancer	recognize m6A modification sites in MYC mRNA in cervical cancer cell lines to promote MYC expression	Enhance cancer cell proliferation, migration, invasion and aerobic glycolysis	(67)
IGF2BP3	Reader	Endometrial cancer	enhance the stability and inhibited the attenuation of E2F3 mRNA	promote the proliferation, migration and invasion of cancer cells	(64)
m^5^C					
NSUN2	Writer	Cervical cancer	Promote m^5^C methylation on KRT13 mRNA and recruit YBX1 to stabilize KRT13 mRNA resulted in increased KRT13 expression	Promote the invasion and migration of cervical cancer cells	(76)
YBX1	Reader	Epithelial ovarian cancer	YBX-1 is degraded by SIAH1-mediated ubiquitination, resulting in instability of target mRNA m^5^C modifications	Enhance the sensitivity of epithelial ovarian cancer to cisplatin and inhibit the proliferation, invasion and migration of cancer cells	(77)
m^1^A					
ALKBH3	Eraser	Ovarian cancer	The half-life of CSF-1 mRNA is prolonged and CSF-1 expression is increased	Enhances the invasion ability of cancer cells	(91)
TRMT10C	Writer	Ovarian and cervical cancer	Unknown	promote the proliferation, colony formation and migration of cancer cells	(92)
A-to-I editing					
ADAR1		Cervical cancer	Unknown	The increased expression of ADAR1 is associated with the malignant progression of cancer cells	(103)
		Ovarian cancer	ADAR1 prevent the accumulation of R ring in ovarian cancer, avoid the DNA damage of cancer cells and the activation of ATR-Chk1 cell cycle checkpoint, so that the cell cycle of G1/G0 phase does not stall.	Promote the growth of cancer cells	(104)
ADAR2		Endometrial cancer	Unknown	Positively correlated with tumor aggressiveness. The increased expression of ADAR2 promote the proliferation and migration of cancer cells and inhibit apoptosis	(105)
Pseudouridine					
DKC1	Writer	Endometrial cancer	Possibly by disrupting normal translation mechanisms	Decreased DKC1 expression was associated with tumor proliferation and low patient survival	(125)
PUS7	Writer	Ovarian cancer	May interact with NOC3L and PUS1 to regulate DNA replication and cell cycle	Promote the proliferation of ovarian cancer	(128)

### 2.2 Eraser

The m^6^A methylation modification process of RNA in the nucleus is dynamic and reversible, and it can be reversed by m^6^A demethylase. FTO (fat-mass and obesity-associated protein) and ALKBH5(a-ketoglutarate-dependent dioxygenase alkB homolog 5) are the two most important m^6^A demethylases currently known. Among them, FTO was the first m^6^A demethylase to be discovered, which is mainly made by Fe (II) and α- Ketoglutaric acid (αKG)-dependent demethylation of m^6^A in substrate mRNA (36). ALKBH5 is a member of the ALKB family as the second known m^6^A demethylase, mainly by making nuclear RNA (mainly m^6^A demethylation on mRNA), thereby affecting nuclear RNA output and metabolism (37). Abnormal expression of FTO and ALKBH5 is closely associated with the occurrence of various diseases, such as leukemia (38), infertility (39), and obesity (40), bladder cancer (41), etc. m^6^A demethylase also plays an important role in gynecological cancers.

FTO acts as an oncogene in cervical cancer. Its demethylation enhances the translation of E2F1 (E2 promoter binding factor 1) and Myc which promotes the proliferation and migration of cervical cancer cells (42). Besides, FTO also plays an oncogene role in endometrial cancer. It can reduce HOXB13 (Germline homeobox B13) mRNA decay by catalyzing the demethylation of HOXB13 mRNA 3’UTR region to eliminate the recognition of m^6^A modification by YTHDF2 protein which increases the expression of HOXB13 protein. Thus, the WNT signaling pathway is activated, and the expression of WNT pathway-related proteins c-myc, snail, MMP2, MMP7 and MMP7 is increased, which enhances the ability of tumor metastasis and invasion (43). However, in ovarian cancer, overexpression of FTO reduces m^6^A levels and mRNA transcript stability which inhibits the hydrolysis of the second messenger cAMP mediated by two phosphodiesterases, PDE1C (phosphodiesterases 1C) and PDE4B (phosphodiesterases 4B). The increase of cAMP level inhibited the ability of ovarian cancer stem cells to self-renew, proliferate and spheroid formation (44). This may be related to the epigenetic reprogramming caused by CAMP-induced PKA activation, which promotes differentiation of tumor stem cells and loss of tumor initiation ability (45).

ALKBH5, another m^6^A demethylase, is highly expressed in human epithelial ovarian cancer (EOC) (46). ALKBH5 demethylated the mRNA of Bcl-2 (B-cell lymphoma-2), which inhibits apoptosis, and increased its stability, thus promoting the interaction between Bcl-2 and Beclin1, inhibiting the autophagy of EOC and promoting the proliferation and invasion of cancer cells. In endometrial carcinoma, demethylase activity of ALKBH5 can inhibit the decay of IGF1R transcript and promote the expression of IGF1R (insulin-like growth factor 1 receptor), thereby inducing the expression of COL1A1(collagen type I alpha 1 chain) and MMP9(matrix metallopeptidase 9) which enhances the proliferation, invasion, and migration of endometrial cells (47).

All these reports indicate that the function of FTO and ALKBH5 in gynecologic cancer mainly depends on the demethylation of m^6^A, and they play an important role in the occurrence and development of gynecologic cancer.

### 2.3 Reader

m^6^A modification of RNA is dynamically and reversibly regulated by methyltransferases and demethylases, while m^6^A recognition protein participates in downstream target gene biology processes by recognizing m^6^A methylation modification information in mRNA. m^6^A recognition proteins are mainly YT521-B homology (YTH) domain-containing family proteins, including YTHDC1 (YTH domain containing 1), YTHDC2 (YTH domain containing 2), YTHDF1 (YTH domain family protein 1), YTHDF2 (YTH domain family protein 2) and YTHDF3 (YTH domain family protein 3). In addition, insulin-like growth factor 2 mRNA-binding proteins (IGF2BPs, including IGF2BP1/2/3), heterogeneous nuclear ribonucleoprotein family (HNRNP family, including HNRNPA2B1/HNRNPC/HNRNPG) and eIF3 (eukaryotic translation initiation factor 3) are also part of the m^6^A recognition protein (48). They can be achieved by influencing RNA degradation (49), translation and decay (50), splicing (51), nuclear transport (52), stability (53) or facilitating the processing of precursor miRNAs (54) plays an important role. m^6^A binding proteins play an important role in the development of a variety of cancers (55–57) by specifically recognizing changes in m^6^A modification levels on target mRNA and regulating specific target genes after modification.

YTHDF2 is highly expressed in human ovarian cancer, and its expression level is positively correlated with the development of ovarian cancer (58). High levels of YTHDF2 can down-regulate the expression of BMF, a pro-apoptotic protein (59), by promoting the attenuation of m^6^A modified transcript, thus promoting the proliferation of ovarian cancer cells, and inhibiting cell apoptosis. On the other hand, in endometrial cancer, YTHDF2 can also promote the degradation of tumor suppressor gene Long noncoding RNA FENDRR, thereby up-regulating the protein expression level of SOX4(SRY-related HMG box transcription factor 4), promoting the proliferation of cancer cells, and inhibiting cell apoptosis (60).

YTHDF1 is also abnormally high expressed in human ovarian cancer. As an oncogene, YTHDF1 can affect the overall protein translation in ovarian cancer cells by promoting the translation and overall output of protein translation initiation factor EIF3C mRNA, thus promoting the proliferation, migration, and invasion of ovarian cancer cells (61).

As another important family member of m^6^A recognition proteins, IGF2BP1, IGF2BP2 and IGF2BP3 are mainly composed of two major domains. The K homology domain is mainly responsible for RNA binding, while the RNA recognition motif domain is responsible for stabilizing the IGF2BP-RNA complex (62). IGF2BP preferentially recognizes m^6^A-modified mRNAs and promotes the stability and translation of the target mRNA in an m^6^A-dependent manner (53). For example, in endometrial cancer, IGF2BP1, by binding to the m^6^A site of SOX2 mRNA 3’UTR, promotes mRNA stability and inhibition of attenuation which enhances the expression of oncogene SOX2(sex-determining region Y-box 2), thus promotes the colony formation, invasion and migration of endometrial cancer (63). In addition, IGF2BP3 can also promote the proliferation, migration, and invasion of cancer cells in human endometrial carcinoma by enhancing the stability and inhibiting the attenuation of E2F3(E2F transcription factor 3) mRNA (64). Recent studies have found that MYC is a key regulator of aerobic glycolysis and is associated with malignant progression of tumors as an oncogene (65, 66). IGF2BP2 can recognize m^6^A modification sites in MYC mRNA in cervical cancer cell lines to promote MYC expression, thereby enhancing cancer cell proliferation, migration, invasion, and aerobic glycolysis (67).

In summary, these proteins usually exist as carcinogenic components in different gynecological cancers, and they are involved in the malignant progression of cancer by affecting the stability, translation, decay, or output and so on of target mRNA by relying on the recognition of m^6^A.

## 3 m^5^C modification in gynecological cancer

**Figure 2:**
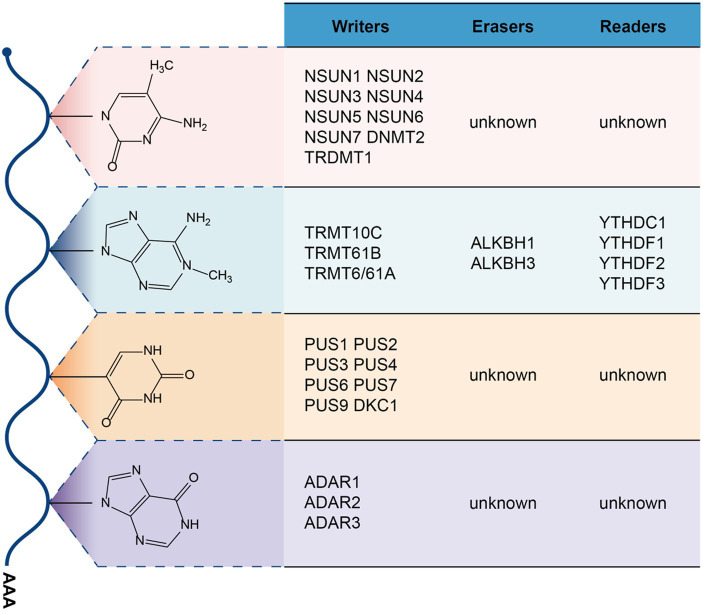
m^5^C, m^1^A, ψ, and A-to-I modifications on RNA.

m^5^C modifications are ubiquitous in RNA, including mRNA, tRNA, and rRNA, and are currently used for DNA or the determination of m^5^C levels in RNA can generally be quantified by bisulfite sequencing (68). A variety of enzymes associated with 5-cytosine methylation modification have been identified, including NOL1/NOP2/sun family (including NSUN1/2/3/4/5/6/7) and DNMT2/TRDMT1 (DNA methyltransferase member 2/tRNA methyltransferase 1), but no clear studies have found enzymes that demethylate m^5^C (69), and m^5^C’s recognition protein is predominantly ALYREF(Aly/REF export factor, an mRNA transport adaptor) (70) and YBX1 (Y-box binding protein 1) (71) (Figure 2). Among m^5^C methylation modification enzymes, NSUN1 and NSUN5 are responsible for m^5^C methylation of cytoplasmic 28S rRNA, NSUN3 and NSUN4 are responsible for methylate mitochondrial tRNA and rRNA, and NSUN7 is responsible for methylate enhancer RNA (eRNA) (72). NSUN2, NSUN6 and DNMT2 are mainly responsible for methylate cytoplasmic tRNA, besides, NSUN2 can also methylate mRNA and vault RNA (VtRNA) (72). In the m^5^C recognition protein, ALYREF mainly recognizes the m^5^C modification site on mRNA edited by NSUN2, which is promoted The mRNA is output from the nucleus to the cytoplasm (70), while YBX1 is output by recognizing m^5^C on the mRNA moddifications thereby recruiting Pabpc1a enhance mRNA stability (71). Recently, many studies have shown that abnormal m^5^C modification promotes cancer proliferation, invasion or metastasis, such as hepatocellular carcinoma (73), bladder cancer (74), Squamous cell carcinoma of the esophagus (75). In gynecologic cancers, abnormalities in the RNA m^5^C modification are also associated with the progression of its malignancy.

Some studies have found that NSUN2 is highly expressed in human cervical cancer tissues, and by enhancing the methylation modification of m^5^C of oncogene KRT13 (keratin 13) mRNA and recruiting YBX1 to further stabilize KRT13 mRNA, the expression of KRT13 is increased, and the invasion and migration of cervical cancer cells are promoted (76). In addition, in epithelial ovarian cancer (EOC), SIAH1 (seven *in absentia* homolog 1), a ubiquitinating ligase, ubiquitinates YBX1 at K304 via the RING domain, making EOC sensitive to cDDP and inhibiting cancer cell proliferation, invasion, and migration (77). It may be that ubiquidization of YBX1 leads to instability of m^5^C modification of E2F5, YY1 and RCC2 mRNAs, thus accelerating the degradation of these target mRNAs.

At present, the research on m^5^C modification in gynecological cancer is very limited, what role other m^5^C modification related enzymes play in the development of gynecological cancer, and whether the potential role of NSUN2 in cancer can be mediated by tRNA modification still need to be further explored and studied.

## 4 m^1^A modification in gynecologic cancer

Modification of m^1^A occurs mainly in cytoplasmic and mitochondrial tRNAs, with 1-methyladenylation usually occurring at positions 58 and 9 of the tRNAs (78, 79). The modification can also occur on mRNA and rRNA. According to current studies, it is known that m^1^A methyltransferases mainly include TRMT10C(tRNA methyltransferase 10 homolog C) (80), TRMT61B(tRNA methyltransferase 61 homolog B) (79), TRMT6/61A(tRNA methyltransferase 6/tRNA methyltransferase 61 homolog A) (81), m^1^A main demethylases are ALKBH1(alkylation repair homolog 1) (82), ALKBH3(alkylation repair homolog 3) (83) and the recognition proteins are mainly YTHDF1-3,YTHDC1 (84). m^1^A modification on tRNA has been shown to promote tRNA structure stability and induce correct tRNA folding (85), and in mitochondrial mRNA, it mainly inhibits mRNA translation through TRMT61B methyltransferase (81). Abnormal expression of m^1^A modification on RNA is also closely related to the pathogenesis of the disease (85, 86).

CSF-1, a macrophage colony stimulating factor, regulates the migration, proliferation, differentiation and survival of macrophages and their precursors by activating its receptor CSF-1R (87). Overexpression of CSF-1/CSF-1R plays an important role in the malignant development of cancer (88–90). In ovarian cancer, ALKBH3 can extend the half-life of CSF-1 mRNA and enhance the expression of CSF-1 by making CSF-1mRNA near the origin of translation 5’UTR region m^1^A demethylation, thereby improving the invasion ability of ovarian cancer cells (91). In addition, TRMT10C is highly expressed in cervical cancer and ovarian cancer tissues, promoting the proliferation, colony formation and migration of ovarian cancer and cervical cancer cells, which may be related to the regulation of C-MYC-related pathways (92).

This evidence supports the important role of m^1^A modification in the development of gynecological cancers, but the information on the key players in the regulation of m^1^A modification is very limited, and there are still unknown potential key enzymes of m^1^A modification that have yet to be discovered.

## 5 A-to-I editing in gynecological cancer

RNA editing events were first identified in the sequence of COXII. transcripts in trypanosomes mitochondria (93). In the animal world, A-to-I RNA editing is the most common, and it is mainly mediated by ADAR enzymes to convert double-stranded RNA (dsRNA) substrate in adenosine is converted to inosine (94). Among them, ADAR1(Adenosine deaminase acting on RNA 1) (95) and ADAR2(Adenosine deaminase acting on RNA 2) enzymes (96) have been shown to have A-to-I editing activity. A-to-I editing modifications in RNA may lead to the production of nonsynonymous codons, leading to the diversity of translation proteins, associated with the development of diseases such as Aicardi-Goutières syndrome (97) and cancer (98, 99). Besides, A-to-I editing works by altering dsRNA in pri-miRNA structure, thereby inhibiting the processing and maturation of pri-miRNA by Drosha (100).

In recent years, the role of A-to-I editing enzymes in gynecological cancers has gradually begun to be discovered. ADAR1 prevents type I interferon in human cells by inhibiting the production of endogenous double-stranded DNA (dsRNA) in the host. The loss of ADAR1 can lead to the accumulation of dsRNA, induce Type I interferon (IFN), promote apoptosis and growth arrest (101). Therefore, in tumors, the high expression of ADAR1 is conducive to the immune escape of cancer cells. Recent studies have also confirmed that the loss of ADAR1 in tumors enhances tumor sensitivity to immunotherapy, thereby overcoming resistance to immune checkpoint blocking (102). In gynecological cancers, ADAR1 mainly acts as an oncogene. In cervical squamous cell carcinoma, the increased expression of ADAR1 is associated with the malignant progression of cancer cells, and its specific molecular mechanism needs to be further studied (103). In addition, ADAR1 is also highly expressed in human ovarian cancer tissues and is inversely associated with progression-free survival. The presence of ADAR1 maintains normal dsRNA editing, prevents R ring accumulation in ovarian cancer, avoids cancer cell DNA damage and ATR-Chk1 cell cycle checkpoint activation, prevents G1/G0 phase cell cycle stagnation, and promotes the growth of cancer cells (104). This study revealed a new ADAR1/R-loop/ATR pathway that is crucial for ovarian cancer progression, and the exploration of ADAR1 inhibitors and ATR inhibitors may be a good direction for future treatment of ovarian cancer.

The mechanism of action of ADAR2 in gynecological cancer has not been well described. Existing studies have found that ADAR2 is overexpressed in endometrial cancer, and its expression level is positively correlated with tumor aggressiveness. The increased expression of ADAR2 promote the proliferation and migration of cancer cells and inhibit apoptosis (105).

Overall, these results demonstrate that adenosine to inosine editing is an important regulatory mechanism in the development of gynecologic cancers, and targeting this pathway, specifically ADAR1, is a promising possibility for gynecologic cancer treatment.

## 6 Pseudouridine nucleoside modification in gynecological cancer

Pseudouridine nucleoside modification occurs after transcription and is one of the most common modifications in RNA (106), and it was first discovered in yeast (107). Pseudouridine nucleoside modification plays an important role in the functional output of tRNA and rRNA. Loss of pseudouridine nuclear modification will lead to decreased expression level of rRNA and enhanced read through activity of stop codon (108), and also affect the translation output of tRNA (109). In addition, pseudouracil nucleosidation modification can also be found in Mt-tRNA, snRNA, miRNA, and lncRNA (110). There is currently no clear research evidence for whether mRNA has naturally occurred pseudouridine modifications, but studies have found that artificially pseudouridation of mRNA can promote non-canonical base pairing at the ribosome decoding center and change the genetic code, resulting in increased protein diversity (111, 112). The main method to identify whether RNA contains pseuduridine modification is the specific binding of pseuduracil nucleoside to n-cyclohexyl-N ‘-(2-morpholine ethyl) carbodiimine methyl-p-toluenesulfonate (CMCT) (113). At present, it is known that enzymes involved in pseudouridine modification mainly include PUS1(pseudouridine synthases 1), PUS2(pseudouridine synthases 2), PUS3(pseudouridine synthases 3), PUS4(pseudouridine synthases 4), PUS6(pseudouridine synthases 6), PUS7(pseudouridine synthases 7), PUS9(pseudouridine synthases 9), DKC1(Dyskerin pseudouridine synthase 1) et al. (113, 114). The abnormalities of their modifier enzymes are involved in straightening the development process of bowel cancer (115), lung adenocarcinoma (116), neurodevelopmental disorders (117), congenital dyskeratosis (118) and other diseases. There are no known erasers or readers for pseudouridine nucleoside modification in eukaryotic cells (119).

Dyskerin protein is a pseudouracil synthetase encoded by DCK1 gene, which can convert specific uracil on ribosomal RNAs (rRNAs), nuclear RNAs (snRNAs) and messenger RNAs (mRNAs) into pseudouracil (120), and participate in many biological processes, including protecting telomere integrity and maintaining ribosomal biogenesis (121). The abnormal expression of Dyskerin is related to the poor prognosis of patients in most cancer research at present (122, 123), and it is considered that Dyskerin maintains high telomerase activity and promotes the proliferation of cancer cells (124). Different from the previous research results, Dyskerin is low in endometrial carcinoma, and the decrease of Dyskerin expression is related to the proliferation of endometrial carcinoma and the low survival rate of patients (125). It is considered that the loss of abnormal Dyskerin may promote the development of endometrial cancer by disturbing the normal translation mechanism (126, 127). In addition, some studies have also found that PUS7 is highly expressed in human ovarian cancer tissues. Through the analysis of gene pathway data, it is considered that PUS7 may interact with NOC3L and PUS1 to promote the proliferation of ovarian cancer by regulating DNA replication and cell cycle (128).

At present, the research progress of pseudouridine modification in gynecological cancer is very limited, and the specific molecular mechanism of pseudouridine synthase in gynecological cancer has not been studied in detail in known studies. Further research is needed to fully explore the role of pseudouridine modification in gynecological cancer in the future.

## 7 RNA-modifying enzyme-related inhibitors and gynecologic cancer

At present, the exploration of small molecule inhibitors of m^6^A has made great progress. In the study of m^6^A methyltransferase inhibitors, some researchers discovered STM2457 as a candidate specific inhibitor catalyzed by METTL3 can cause damage to AML mouse model modeling and prolong the survival time of mice (129). In addition, through drug library screening, quercetin can also act as an inhibitor of METTL3 to inhibit the proliferation of pancreatic and liver cancer cells (130). A newly synthesized compound, UZH2(lead compound 22) (131), has also been shown to have the inhibitory activity of the METTL3 enzyme. Inhibitors of m^6^A demethylase, such as meclofenamic acid (MA) (132), Rhein (133), R-2HG (134), FTO-04 (135), FB23 and FB23-2 (136), have been discovered in recent years. They all have FTO enzyme inhibitory activity and can be used in AML (134, 136), Glioblastoma (135) and other cancers have shown significant antitumor activity. MV1035 (137) and ALK-04 (138) as inhibitors of ALKBH5 also show tumor suppressor effects in glioblastoma and melanoma. For inhibitors of the m^6^A-recognition protein, a compound called “7,773” has been found to bind IGF2BP1 and inhibit its interaction with KRAS RNA (139), thereby reducing the expression of KRAS protein and downstream signaling, inhibiting the cancer-promoting activity of IGF2BP1. BTYNB (140) inhibited the malignant process of ovarian cancer by weakening the stability of IGF2BP1 on E2F1 mRNA, thus limiting the protein translation of E2F1. MO-460 (141), a newly synthesized similar based on (R)-(−)-Moracin-O, acts on the glycine-rich C-terminal domain of hnRNPA2B1 and inhibits binding of hnRNPA2B1 to the 3′-untranslated region of HIF-1αmRNA. The inhibition of HIF-1α translation can play an anticancer role.

As for inhibitor studies of other RNA-modifying enzymes, RUS0207-A006, RUS0202-G005 and JK0395-B007 have been found to have inhibitory activity of YBX1 recognition protein (142), and RUS0207-A006 and JK0395-B007 inhibited malignant melanoma cells, Proliferation of colon cancer cells and breast cancer cells. In addition, azacytidine (143) has also been found to act as an inhibitor of the RNA methyltransferase DNMT2, inhibiting the main substrate of DNMT2, tRNA (Asp) Methylation of the C38 site. and 2,3-diaryl indenones (144) as ALKBH3, a selective inhibitor that plays a significant inhibitory role in the proliferation of lung cancer cells.

More and more RNA-modifying enzyme-related inhibitors are gradually being synthesized or discovered, and their anti-cancer effects in a variety of tumors are gradually being shown to us, but their specific tumor suppression mechanisms need to be further explored.

## 8 Future perspectives

With the discovery and functional study of RNA modification types and various RNA modification-related regulators, the understanding of RNA modification has been greatly improved. Despite tremendous progress, there are still many unknowns about our understanding of RNA modifications and their specific regulatory mechanisms in gynecologic tumors. From a macro perspective, many of the authors, readers, and erasers of modifications are still unknown, which hinders further research into the functional mechanisms of these modifications. In addition, the mechanisms by which many known writers, readers, and erasers can selectively identify, install, or remove modifications, as well as the mechanisms by which their specific environments and spatiotemporal manipulations are regulated, remain unknown. The full range of regulatory and biological roles of mRNA modification remains largely unexplored. In some cases, one enzyme produces opposite effects in different cancer types, or even in the same cancer type. Given the diversity of RNA modifications and the sheer number of modified coding RNAs and ncrRNAs, it may not seem surprising that this contradiction sometimes arises. Therefore, more relevant studies are needed to further validate and explain the specific mechanism of RNA methylation in cancer, and to explain more rationally some of the existing conflicting studies. In addition, RNA modifications have greatly contributed to the development of therapeutics, including antisense oligonucleotides, RNA aptamers, and short-interfering RNA drugs. Given the great success of Ψ-modified mRNA vaccines for infectious diseases, the development of modified vaccines for the treatment of cancer has bright prospects. In addition, we believe that the development of small molecule inhibitors targeting RNA modification sites and RNA-modifying enzymes is very promising, which will provide a new and more targeted approach to cancer treatment. Specific to the field of gynecologic oncology, we believe that there are still many issues that need to be clarified: (1) the changes of RNA modification in different stages of gynecological cancer development are not clear; (2) Does the same RNA modification between different types of RNA (mRNA, rRNA, tRNA, lncRNA) produce different biological functions? (4) The carcinogenic and tumor suppressor functions of RNA modification regulators sometimes play opposite roles in different cancers, and their complex regulatory mechanisms need to be further studied; (5) it would be interesting to elucidate crosstalk or competition between different types of RNA modifications; (6) Whether the changes in RNA modification can be used as a predictor of treatment response in patients with gynecologic tumors needs to be studied; (7) Although drugs targeting RNA-modified enzymes have shown potential to promote the efficacy of chemotherapy or immunotherapy in preclinical studies, related research is still in its infancy, and rigorous clinical trials are needed to prove their efficacy in the future. (8) The prediction of prognostic RNA modification-related molecules is often based on bioinformatics and database mining, and further experimental verification and prospective clinical trials are needed.

## Author contributions

WH: Writing – original draft. XH: Conceptualization, Writing – original draft. GC: Data curation, Writing – original draft. XL: Investigation, Writing – review & editing. YL: Conceptualization, Investigation, Writing – review & editing.
